# The long and winding road: the journey taken by headache sufferers in search of help

**DOI:** 10.1017/S1463423618000324

**Published:** 2018-05-31

**Authors:** Paul T.G. Davies, Russell J.M. Lane, Theresa Astbury, Manuela Fontebasso, Jill Murphy, Manjit Matharu

**Affiliations:** 1 Department of Neurology, Northampton General Hospital, Billing Road, Northampton, UK; 2 Consultant Neurologist, Northampton General Hospital, Northampton, UK; 3 Honorary Senior Lecturer, Clinical Neurology Radcliffe Infirmary, Oxford, UK; 4 Department of Neurology, Ashford Hospital, Middlesex, UK; 5 York Headache Clinic (York District Hospital), York, UK; 6 The London Headache Group, National Hospital for Neurology and Neurosurgery, Queen Square, London, UK

**Keywords:** chronic migraine, headache clinic, headache management, headache pathway, medication overuse headache

## Abstract

**Aim:**

To outline the pathways a cohort of first attendees to our headache clinics had taken over the years in search of explanations and treatment for their headaches. To establish a greater awareness of the shortcomings and failures in their medical journey in the hope that better headache management will emerge in primary care.

**Background:**

At first attendance in primary care most headache sufferers will not receive a firm diagnosis. Treatments provided are often ineffective and so many patients embark on a somewhat random self-made journey searching for a remedy. If they reach a Headache Clinic the most common diagnoses are ‘chronic migraine’ and ‘medication overuse headache’. They are either no better or worse than when their headaches first started despite their efforts.

**Method:**

We undertook a prospective questionnaire-based study of over 200 patients on first attendance at each of our headache clinics, three based in District General Hospitals and one in a tertiary referral centre. We documented the patients’ headache characteristics, the ‘burden’ of their headaches, functional handicap and the financial costs incurred seeking help before referral. We also documented what our patients understood about their headache disorder and the treatments previously tried.

**Findings:**

Most patients had not been given a formal diagnosis in primary care and many remained unconvinced of the benign nature of their headache problem and wanted further investigations. A few had sought help from headache charities. Many had unrealistic attitudes to their problem and medication overuse was rife. A few patients had been offered triptans in primary care. Key deficiencies in the primary care management of these patients included failure to provide a formal headache diagnosis, inadequate understanding of the nature and mechanism of headaches and failure to follow a resilient management strategy. We provide a more effective management pathway in primary care.

## Introduction

Headache is the commonest of all symptoms and probably a universal experience at some time of life. Primary headache syndromes, notably ‘migraine’ and ‘tension type headache’ as defined by the International Headache Classification [Headache Classification Committee of the International Headache Society (IHS), 2013a; 2013b] affect more than 80% of the adult population in the United Kingdom; 70% of people will experience a significant headache within a three-month period with 20% reporting missed or reduced ability work days as a result (Steiner *et al*., 2003).

It is not therefore surprising that headache is a frequent presenting symptom in primary care. About 3% of the general population consults primary care for headache each year making it the most common neurological presentation (Latinovic *et al*., 2006). ‘Chronic headache’ [chronic daily headache (CDH)], defined under the IHC as attacks of headache occurring on more than half the days in a month over a period of more than three months affects 2–4% of the UK population (Stovner *et al*., 2007). CDH is very often ‘chronic migraine’, which nearly always develops from episodic migraine (Bigal *et al*., 2008) and nearly half of such cases also have potentially avoidable medication overuse headache (MOH) (Castillo *et al*., 1999).

In worldwide terms headache is the third most common cause of disability (Steiner *et al*., 2015). Headache also has a significant social and economic impact. According to the World Health Organisation, migraine is the fifth most common cause of ‘years lived with disability’ (WHO, 2012), ahead of diabetes and osteoarthritis (Vos *et al*., 2013), and the annual cost of headache disorders in the United Kingdom is estimated to be £5–7 billion (Steiner, 2010).

If correctly diagnosed, nearly all headache patients can be managed in primary care, where common headache co-morbidities and the complex psychosocial factors that are often important in the provocation and perpetuation of headache can be addressed most effectively (The Association of British Neurologists,^1993^; Belam *et al*., 2005; Ridsdale and Kernick, 2010). However, most adult migraineurs have never consulted their general practitioner despite high levels of disability (Thomas *et al*., 2004) and only 2% of those seen in primary care are referred to a neurologist (Latinovic *et al*., 2006). Of those who do present to their GP, 70% will receive no formal diagnosis and it has been suggested that GPs’ difficulties in diagnosing primary headache contribute to its morbidity and unmet need (Kernick *et al*., 2008). In Kernick’s study only 5% received a diagnosis in the first year after consultation and in 73% the diagnosis was ‘migraine’ (Kernick *et al*., 2008).

For many headache patients, pharmacists and opticians are alternative sources of advice and treatment and many patients self-manage (Peters *et al*., 2003). Others attend Accident and Emergency departments where migraine is the most common of all neurological presentations (Gahir and Larner, 2006).

Very little is known of the journeys patients take before reaching one of the relatively few headache neurologists or specialist headache clinics in the United Kingdom. The authors all work in secondary or tertiary care and specialise in headache. When comparing our practices, we realised we were seeing similar ‘problem’ headache patients – mostly middle age women with long standing chronic migraine, usually previously undiagnosed and often complicated by MOH. Many were also taking migraine prophylactic medications that were clearly ineffective and had also seen a variety of healthcare practitioners with various qualifications in the search for a solution to their headache problem.

We had no reason to believe that patients seen in other UK headache clinics would differ significantly from ours. We therefore decided to survey the patients referred to our practices using a questionnaire that we devised to see if our impressions were confirmed and if so, whether a more productive approach could be recommended. In particular, we examined the patients’ headache characteristics and the ‘burden’ of headache in terms of chronicity, functional handicap and financial costs incurred seeking a remedy before referral. We also documented what our patients understood about their headache disorder and the treatments they had tried previously.

## Methods

We conducted a six-month survey of consecutive new patients referred to specialist headache clinics at three district general hospitals (Northampton, York, and Ashford, Middlesex) and one tertiary referral centre [National Hospital for Neurology and Neurosurgery (NHNN), Queen Square, London]. Patients were invited to complete a ‘Headache Pathway’ questionnaire (see Appendix). In addition to demographic data, we documented: how long patients had suffered a headache problem before consulting a GP; the number of GP attendances before referral for expert opinion, time to referral, and whether they had episodic or chronic headache. We also recorded whether patients had been given a specific headache diagnosis by their GP and whether they wanted a brain scan and if so, why. We also asked which other health professionals they had consulted for headache management, how much money they estimated to have spent addressing their headache problem, and how much time off work or full-time education they had lost because of headaches. We documented the headache treatments they had received before referral and how effective they had been, and whether they knew of any of the UK headache charities.

## Results

A total of 209 questionnaires were completed (Northampton 46, York 43, Ashford 80 and NHNN 40). The respondents comprised 199 women and 10 men mean age 44.3±15.6 (range 17–82 years). When respondents did not provide an answer to a question, this was recorded.

### Headache characteristics and pre-referral diagnosis

More patients had chronic than episodic headaches by a ratio of about 2:1 except in Northampton, where there were more patients with episodic headache. Of 200 responders, only 72 (36%) had been given a specific headache diagnosis before referral. Some patients underwent brain scanning via our headache clinics as a result of patient demand but no relevant intracranial abnormalities were found. Following assessment, all the patients were considered to fulfil criteria for the defined primary headache disorders, ‘episodic migraine’ or ‘CDH’, now considered to be ‘chronic migraine’ (CM), sometimes complicated by MOH (IHS, 2013a) (Table 1).

**Table 1:** Characteristics of patients’ headaches and pre-referral diagnosis

	Northampton	York	Ashford	NHNN
No. of patients with episodic or chronic daily headache	16 Episodic	No data	15 Episodic	9 Episodic
	14 Chronic		29 Chronic	30 Chronic
	16 No response		30 No response	1 No response
No. of patients with a pre-referral headache diagnosis	15 Diagnosed	20 Diagnosed	22 Diagnosed	15 Diagnosed
	26 No diagnosis	21 No diagnosis	57 No diagnosis	24 No diagnosis
	5 No response	2 No response	1 No response	1 No response

NHNN=National Hospital for Neurology and Neurosurgery.

### Headache burden

#### Headache history and visits to GPs

Patients seen in the DGH clinics reported having headaches for between 6 and 10 years on average before referral. The mean duration was still longer (13.5 years) at the tertiary referral centre (NHNN). All patients reported consulting their GP on numerous previous occasions for headache management before referral. The lowest average number of visits was in York (8.5) and the highest at NHNN (14.8) (Tables 2 and 3).

**Table 2:** Headache years and number of GP visits before referral

	Northampton	York	Ashford	NHNN
Duration of headache problem before referral (years)	*n*=44 (2)	*n*=43 (0)	*n*=75 (5)	*n*=40 (0)
	8.6±8.8	10.3±14.1	6.4±9.6	15.4±13.5
Mean number of visits to GP before referral	*n*=41 (5)	*n*=39 (3)	*n*=59 (22)	*n*=35 (7)
	11.3±11.2	8.53±8.7	9.3±15.3	14.8±9.4
	Mode=10	Mode=8.7	Mode=3	Mode=10

The number of patients who did not provide data is shown in brackets. NHNN=National Hospital for Neurology and Neurosurgery.

**Table 3:** Time lost from work or full-time education because of headache and private treatment costs

	Northampton	York	Ashford	NHNN
In paid employment/full time education	*n*=44 (2)	*n*=39 (4)	*n*=73 (7)	*n*=40 (0)
	33	24	52	15
Days lost in last 12 months?	Mean=13.8±17.7	Mean=6.8±8.8	Mean=21.9±57.3	Mean=43.7±98
Estimated self-financed treatment costs	34 (12)	19 (23)	56 (24)	31 (9)
	£354±382	£612±1174	£421±2777	£1935±827

Figures in brackets indicate numbers of non-responders.

#### Time lost from work and full-time education

A total of 124/196 respondents (63%) were in work or full-time education. Table 3 shows the headache ‘burden’ in terms of time lost from work or education because of headache in the 12 months before survey. This ranged from an average of about 14 days in Northampton to 44 at NHNN.

#### Private treatment costs

In addition to seeking help through the NHS, many patients had paid for private treatment. Most had spent hundreds of pounds over the years (Table 3). The most common privately funded treatments were changes in glasses prescriptions and ‘alternative therapies’ (see below).

### Patients’ understanding of their headache problem

Most patients (64%) had not been given a headache diagnosis by their GP. At NHNN, 40% knew that their diagnosis was ‘chronic migraine’. Of the 66 DGH clinic patients who provided responses, 72% wanted a brain scan. This proportion was much less at the tertiary centre (only 2 of 31 respondents) presumably because many had been scanned previously in primary or secondary care. The reasons given for wanting a brain scan varied. Some said ‘to find the cause of my headaches’, or ‘to get to the bottom of the problem’; some ‘because I’m worried’ or ‘for reassurance’. Some did not know why they wanted a scan and some patients who had been scanned previously wanted another scan (Table 4).

**Table 4:** Pre-referral diagnosis and requests for brain scan

		Northampton *n*=46 (5)	York *n*=43 (2)	Ashford *n*=80 (1)	NHNN *n*=40 (1)
Diagnosis given before referral?	Yes	15	20	22	15
	No	26	21	57	24
Brain scan wanted?	Yes	16	No data	31	2
	No	10		9	29

Figures in brackets indicate number of non-responders.

### Previous sources of medical help

In addition to consulting GPs on numerous occasions before referral, the majority of patients at all centres had also attended a wide range of other professionals, and some several. 62% had consulted an optician (Table 5).

**Table 5:** Other practitioners consulted

	Northampton	York	Ashford	NHNN
Optician	29 of 46 (63%)	31 of 43 (72%)	54 of 89 (60%)	29 of 41 (71%)
Physiotherapist	5	3	4	5
Osteopath	3	4	3	10
ENT clinic	1	1	6	8
Chiropractor	3	1	6	12
Dentist	7	2	19	14
Acupuncturist	8	0	10	15

Across the four centres less than 15% of patients were aware of the major UK Headache charities (The Migraine Trust and Migraine Action) which can be consulted for management advice and support. In Northampton, only five patients knew of a headache charity (35 did not; six no answer); in York five were aware (30 were not; seven no answer), and at Ashford just four patients knew of these charities (70 were not; six no answer). However, at NHNN, 19 patients were aware of the headache charities (17 were not; four no answer).

### Previous drug treatment and treatment responses

Patients in all centres had tried a wide variety of analgesics to treat their headaches. The most commonly used was paracetamol, followed by ibuprofen and aspirin. Disconcertingly, nearly all patients had used Co-codamol and other opiate containing drugs frequently, regularly and often at high doses at some time. Although triptan use was quite common the reported treatment response was not impressive. The most frequently used prophylactic drugs were amitriptyline and propranolol followed by sodium valproate, topiramate and pizotifen. The number of patients who became pain free after taking their acute treatment and the number who had previously tried prophylactic drug treatment is summarised in Table 6.

**Table 6:** Use and response to acute treatment, including triptans, and previous use of migraine prophylactic drugs

	Northampton (46)	York (44)	Ashford (82)	NHNN (42)
Triptans	22 Tried	9 Tried	22 Tried	23 Tried
	13 Not tried	12 Not tried	41 Not tried	0 Not tried
	11 No answer	23 No answer	19 No answer	19 No answer
Pain free (more times than not) after using current acute treatment	10 Pain free	13 Pain free	11 Pain free	4 Pain free
	35 Not	27 Not	69 Not	35 Not
	1 No answer	3 No answer	2 No answer	3 No answer
Prophylactic treatment	30 Tried	9 Tried	22 Tried	23 Tried
	14 Not tried	12 Not tried	41 Not tried	0 Not tried
	2 No answer	23 No answer	19 No answer	19 No answer

## Discussion

Our study suggests that in England, the headache sufferer’s journey in search of explanation and treatment can be a long and frustrating one. Most patients referred to our expert clinics were middle aged women with frequent episodic headaches or CDH. ‘Migraine’ or ‘chronic migraine’, often complicated by medication overuse, was the final diagnosis in almost all. Many of these patients had suffered headaches for a decade or more before referral and as a result had lost significant periods of time from work or full-time education.

Despite numerous previous consultations with their GPs, only a third of the patients had been given a specific headache diagnosis before referral. This must surely have resulted in sub-optimal or incorrect treatment in many cases. In our experience, the first advice given for troublesome headaches in primary care is usually an assessment by an optician, although primary ophthalmic problems such as refractive errors are very rarely a cause of headaches in isolation (IHS, 2013b). Migraine in primary care is also often wrongly attributed to ‘sinusitis’ and treated as such (Al-Hashel, 2013).

Without a correct diagnosis, patients can have little understanding as to the true nature of their headache problem and its mechanisms and this is likely to not only heighten anxieties as to causation but impede effective management.

Although no pathological cause for headache was suspected or found in any patient in our study, many patients were anxious about undetected disease despite the chronicity of their headaches; 72% wanted a brain scan, some even if they had been scanned previously. Many patients thought this would ‘get to the bottom of the problem’ but there is no indication for such investigation in the vast majority of cases according to the many publications on ‘when to scan headache’ (Ahmed, 2012). Scanning headache patients in primary care may reduce referrals to secondary care but rarely provides a diagnosis and is unlikely to help the longer-term management in primary care. Indeed, it may add to their anxieties (Howard *et al*., 2005). Packard concluded that in general, physicians overestimate patients’ wish for ‘headache treatment’ and underestimate their need to understand the basis of their headaches. More emphasis on diagnosis and explanation is clearly needed (Packard, 1979).

Although migraine was by far the most common diagnosis in our cohort, less than half the DGH patients had been offered triptans. In addition, only a minority enjoyed a satisfactory response to their acute treatment. Many actually had undiagnosed chronic migraine and prophylactic therapy should have been a key intervention in management. Although prophylactic drugs had been prescribed in a minority the treatment regimes had been largely ineffective. Many patients had therefore become over-reliant on analgesics. It is well recognised that analgesic overuse is a significant factor in headache chronicity and a frequent complication among problem headache clinic patients (Dowson, 2003; Meskunas *et al*., 2006). Opiates and compound analgesics are especially culpable and it was sobering to find that most patients in our study had used Co-codamol and other opiate based drugs frequently and on a regular basis despite widely published advice on the dangers of these drugs as a treatment for headache. BASH states that opiates and opioids (including diamorphine, morphine, pethidine, dextropropoxyphene, buprenorphine, tramadol, codeine and dihydrocodeine) ‘should be avoided’ in acute migraine treatment (BASH, 2010).

It is not surprising therefore that our patients looked to alternative resources for help. In addition to opticians, many had also sought the advice of physical therapists (physiotherapists, osteopaths and chiropractors) and had received treatment based on the concept of ‘neck therapies’ although there is little evidence for their effectiveness in the headache population (Biondi, 2005). Although we did not enquire how our patients had made their choices of alternative practitioners, patients had spent considerable amounts of money without benefit.

The majority of our patients had not consulted any of the UK headache charities for advice. These organisations provide excellent evidence-based information not only for conditions like migraine but also with regard to the availability of headache services throughout the United Kingdom.

In summary, our study showed high levels of unmet need and significant economic impact from primary headaches. Most headaches are benign but frequent attacks can be intrusive and disabling. Episodic and chronic migraine are by far the most common headache semiologies but a formal diagnosis at the outset supported by authoritative evidence is an essential part of management. There are many readily available evidence-based guidelines for headache management (British Association for the Study of Headache, 2010; Scottish Intercollegiate Guidelines Network. Diagnosis and management of headache in adults, 2008; National Institute for Health and Care Excellence, 2012), and chronic migraine (Weatherall, 2015). In our opinion, more frequent use of triptans instead of analgesics for episodic headaches and the early introduction of prophylactics with regular review and supervision during dose escalation at an early stage might reduce the tendency to develop chronic migraine and MOH. In addition, there is evidence that improved education for general practitioners supported by general practitioners with a special interest in headache (GPwSI) has the potential to significantly reduce the burden of headache in the United Kingdom. GPwSIs reduce costs as they order less head scans than non-specialist neurologists (Elliot and Kernick, 2011). A healthcare system incorporating GPwSI has been described (Steiner *et al*., 2011). Improvements in the management of headache in primary care in the United Kingdom are needed now to not only treat the type of patients we see most often in our headache clinics but to try and prevent the common complications that so often develop when the nature of headache is misunderstood and mismanaged.

## Conflicts of Interest

This research received no specific grant from any funding agency, commercial or not-for-profit sectors.

## Ethical Standards

Ethical approval for this survey was granted by the Chairman of the Oxford Regional Ethics Committee.
